# Reviewed article: Alberti AM, Silva PBE, Azambuja FS, Godoy L, Concolatto FP, Lech GE, Stein AT. Epidemiological profile of surgical treatment of lung cancer in Brazil: retrospective analysis (2014-2023). Epidemiol Serv Saude. 2025:34;e20240427

**DOI:** 10.1590/S2237-96222025v34e20240427.b

**Published:** 2025-08-04

**Authors:** Oswaldo Villa

**Affiliations:** USP, Periodontics

**Table:** 

Rating	Excellent	Good	Average	Below average	Poor
Interest		✓			
Quality			✓		
Originality			✓		
Overall			✓		

**Table:** 

Questionnaire	Yes	No	Not applicable
Does the manuscript contain new and significant information to justify publication?		✓	
Does the Abstract (Summary) clearly and accurately describe the content of the article?		✓	
Is the problem significant and concisely stated?	✓		
Are the methods described comprehensively?	✓		
Are the interpretations and conclusions justified by the results?		✓	
Is adequate reference made to other work in the field?		✓	

## Manuscript Structure

Length of article is: Too long

Number of tables is: Adequate

Number of figures is: Adequate

Please state any conflict(s) of interest that you have in relation to the review of this paper (state “none” if this is not applicable):

I dont have a conflict of interest.

## Comments

Orientações gerais do manuscrito intitulado: Perfil epidemiológico do tratamento cirúrgico do câncer de pulmão no Brasil: Uma análise de dez anos.

Resumo.

1) Os métodos descritos no resumo não precisa colocar os testes estadísticos, e na parte dos resultados carece de dados importantes de estadística como (%) das diferencias o os achados. Melhore a escrita elimine dados incensários e acrescente os importantes.

Método e estadística:

1) descreva os métodos de seleção de participantes e as variáveis com mais detalhes.

2) Justifique a escolha dos testes estatísticos e explique os resultados de forma clara.

Resultados

1) Apresentar resultados em sequência que evolua linearmente seguindo a ordem das ilustrações;

2) no segundo paragrafo dos resultados explica a : figura 2A e segue em sequência correta mas.: a tabela 1 não e clara e não e autoexplicativa os dados incluídos devem ser autoexplicativos.

3) As tabelas e figuras precisam ser revisadas para garantir clareza, precisão e evitar redundância com o texto.

4) Evitar extrapolações ou interpretações dos resultados (que pertencem à discussão).

Discussão

1) Destaque a originalidade do trabalho, relacionando-o com a literatura existente e explicando sua contribuição para o campo de estudo.

2) O primeiro parágrafo da discussão deve resumir os achados, interpretando-os e sem repetir números nem citar ilustrações: não informar o que fez, interpretar o que encontrou.

3) Apresentar as limitações do estudo, levando em considerações fontes potenciais de viés ou imprecisão no segundo parágrafo.

4) A formatação da discussão não segue a linha da raciocino, interpretar cada resultado relevante e discuti-lo a luz da literatura.

5) Evitar parágrafos sem conexão com a literatura ou que somente a revisam sem discussão dos resultados.

6) recomendo refazer a discussão em base aos guidelines.

